# Untangling Nucleotide Diversity and Evolution of the H Genome in Polyploid *Hordeum* and *Elymus* Species Based on the Single Copy of Nuclear Gene DMC1

**DOI:** 10.1371/journal.pone.0050369

**Published:** 2012-12-10

**Authors:** Dongfa Sun, Genlou Sun

**Affiliations:** 1 College of Plant Science and Technology, Huazhong Agricultural University, Wuhan, People's Republic of China; 2 Biology Department, Saint Mary's University, Halifax, Nova Scotia, Canada; Nanjing Agricultural University, China

## Abstract

Numerous hybrid and polypoid species are found within the Triticeae. It has been suggested that the **H** subgenome of allopolyploid *Elymus* (wheatgrass) species originated from diploid *Hordeum* (barley) species, but the role of hybridization between polyploid *Elymus* and *Hordeum* has not been studied. It is not clear whether gene flow across polyploid *Hordeum* and *Elymus* species has occurred following polyploid speciation. Answering these questions will provide new insights into the formation of these polyploid species, and the potential role of gene flow among polyploid species during polyploid evolution. In order to address these questions, disrupted meiotic cDNA1 (DMC1) data from the allopolyploid StH *Elymus* are analyzed together with diploid and polyploid *Hordeum* species. Phylogenetic analysis revealed that the H copies of DMC1 sequence in some *Elymus* are very close to the H copies of DMC1 sequence in some polyploid *Hordeum* species, indicating either that the H genome in theses *Elymus* and polyploid *Hordeum* species originated from same diploid donor or that gene flow has occurred among them. Our analysis also suggested that the H genomes in *Elymus* species originated from limited gene pool, while H genomes in *Hordeum* polyploids have originated from broad gene pools. Nucleotide diversity (π) of the DMC1 sequences on H genome from polyploid species (π = 0.02083 in *Elymus*, π = 0.01680 in polyploid *Hordeum*) is higher than that in diploid *Hordeum* (π = 0.01488). The estimates of Tajima's D were significantly departure from the equilibrium neutral model at this locus in diploid *Hordeum* species (P<0.05), suggesting an excess of rare variants in diploid species which may not contribute to the origination of polyploids. Nucleotide diversity (π) of the DMC1 sequences in *Elymus* polyploid species (π = 0.02083) is higher than that in polyploid *Hordeum* (π = 0.01680), suggesting that the degree of relationships between two parents of a polyploid might be a factor affecting nucleotide diversity in allopolyploids.

## Introduction

Hybridization and polyploidization have played a central role in the history of plant evolution, and contributed greatly to plant diversification and speciation [1], [2]. Much attention has been drawn to studying the evolutionary consequences of polyploid species in both genome size and contents, with the advances in molecular methods over the last two decades [3], [4]. Polyploid genome origins and evolution have also been the focus of plant evolutionists [1], [5]. Increasing evidences have demonstrated the complexity of the dynamic nature of polyploids. Many polyploids are proved to involve multiple origins in space and time [1], [5], together with introgression (or gene flow) [6]–[8]. Both multiple origins [9], [10] and gene flow [6]–[8] have been considered as the causes of shared polymorphism across ploidy level and/or phylogenetic incongruence among loci. However, whether gene flow among independent formations is regular occurrence following polyploid species have rarely been tested in ployploid taxa [11].

The tribe Triticeae contains several important cereal crops such as wheat, barley and rye, as well as forage crops. The tribe combines a wide variety of biological mechanisms and genetic systems which makes it an excellent group for research in evolution, genetic diversity, taxonomy, and speciation in plants [12]. According to Löve [13] and Dewey's [14] classification, genus *Hordeum* and *Elymus* are two relative large genera in the tribe Triticeae.

The genus *Hordeum* comprises 31 species (including cultivated barley, *H. vulgare* ssp. *vulgare*) and exists at the diploid, tetraploid, and hexaploid levels with a basic chromosome number x = 7. Based on cytogenetic analyses, the diploid species in *Hordeum* were classified into four monogenomic groups: **H, I**, **Xa**, and **Xu** genome group [15], [16], which were supported by isoenzyme analysis [17] and molecular data [18]–[22]. The **H** genome group is not only the largest genome group in *Hordeum* (including 14 diploid species, 7 tetraploid species, 4 hexaploid species, and 2 species existing at three ploidy levels (2×, 4×, 6×), and distributed widely from central Asia to the Americas), but also widely present in polyploid species in *Elymus*, *Stenostachys* and *Pascopyrum*.

Within the genus *Elymus* are approximately 50 allotetraploid species that combined both H and St genomes, and distributed throughout the world in non-tropical areas, from northern Greenland in the north to Tierra del Fuego in southernmost South America [23]. The St haplome originated from the genus *Pseudoroegneria* [14]. It has been confirmed that the **H** haplomes in *Elymus* were contributed by different *Hordeum* diploids [24]–[30]. Phylogenetic analyses based on phosphoenolpyruvate carboxylase, β-amylase, granule-bound starch synthase I [29] and disrupted meiotic cDNA (DMC) [30], suggested few potential *Hordeum* diploid species as **H**-genome donors to *Elymus* species. The tetraploid *H. jubatum* might have involved in the origin of StH *Elymus* [29]. However, the role of polyploid *Hordeum* species in the origin of StH *Elymus* remains to be studied. It is not clear whether gene flow across polyploid *Hordeum* and *Elymus* species has occurred following polyploid speciation. Recent studies led to the conclusion that the polyploid probably originated multiple times [1], [5], which are often considered as a potential source of increased genetic variation in polyploids. However, how much genetic variation is contributed by the diploid progenitors and the degree of gene flow among the independent origins are the two major factors determining the genetic diversity in polyploids. Yet, the extent and role of gene flow among polyploids in evolution remains enigmatic.

In present study, DMC1 data from the allopolyploid StH *Elymus* are analyzed together with diploid and polyploid *Hordeum* species. The objectives of this analysis are: (1) to explore the possible role of polyploid *Hordeum* species in the origin of StH *Elymus*; (2) to determine whether gene flow has occurred between polyploid *Hordeum* and *Elymus*; and (3) to examine the level of nucleotide polymorphism in the H genomes from *Elymus*, *Hordeum* diploids and polyploids. Answering these questions will provide new insights into the formation of these polyploid species, and the potential role of gene flow among polyploid species during polyploid evolution.

## Materials and Methods

### Samples

The present study includes 18 tetraploid (22 accessions) StH *Elymus*, 9 polyploid *Hordeum* species. All diploid *Hordeum* species and other diploid Triticeae species representing the **St**, **W**, **P**, and **E** genomes were included for analyses (Table 1). *Bromus arvensis* and *B. sterilis* were used as outgroups. The single copy nuclear gene disrupted meiotic cDNA (DMC1) has been applied to phylogenetic analyses in Triticeae species. The DMC1 sequences used in this study were collected from previously published sources [21], [30]–[35].

**Table 1:** Taxa used in this study.

Species	Ploidy	Accession no.	Genome	Origin*	GenBank accession no.	Authors
*Agropyron cristatum* Gaertn.	2×	H4349	**P**	Turkey	AF277241	Petersen & Seberg, 2000
*Australopyrum retrofractum* (Vickery) Á. Löve	2×	H6723	**W**	Australia	AF277251	Petersen & Seberg, 2000
*Bromus arvensis* L.		C618		NA	DQ247821	Petersen *et al.*, 2006
*Bromus sterilis* L.		OSA420		Denmark	AF277234	Petersen & Seberg, 2000
*Elymus canadensis* L.	4×	PI 531567	**StH**	Alberta, Canada	EU366405, EU366406	Sha et al. 2009
*Elymus caninus* (L.)L	4×	H3169	**StH**	Västmanland, Sweden	H3169L, H3169k	Sun & Zhang, 2011
		PI314621	**StH**	NA	EU366407, EU366408	Sha et al., 2009
*Elymus confusus* E. Desy	4×	PI 598463	**StH**	Russian Federation	598463k	Sun & Zhang, 2011
		W6 21505	**StH**	NA	GQ855188	Wang et al., unpublished
*Elymus cordilleranus* Davidse &Pohl	4×	H6486	**StH**	Cajamarca, Peru	H6486k, H6486Y	Sun & Zhang, 2011
*Elymus dentatus* (Hook. F.) Tzvelev	4×	PI 628702	**StH**	Altay, Russian	PI 628702	Sun & Zhang, 2011
*Elymus fibrosus* (Schrenk) Tzcelev	4×	H10339	**StH**	Pelkosniemi, Finland	H10339K	Sun & Zhang, 2011
*Elymus gayanus* Desv.	4×	W6-13828	**StH**	Santa Cruz, Argentia	W6-13828L,W6-13828K	Sun & Zhang, 2011
*Elymus hystrix* L.	4×	H5495	**StH**	Canada	H5495R, H5495K	Sun & Zhang, 2011
	4×	PI531616	**StH**	NA	EU366415, EU366416	Sha et al. 2009
*Elymus lanceolatus* (Scribn. & Smith) Gould	4×	PI 236663	**StH**	Maryland, United States	PI 236663K	Sun & Zhang, 2011
*Elymus multisetus* (JG Sm.) Burtt Davy	4×	W6-20963	**StH**	California, United States	W6-20963Y, W6-20963R	Sun & Zhang, 2011
*Elymus sibiricus* L.	4×	PI 619579	**StH**	Xinjiang, China	GQ855198, EU366409	Sha et al. 2009
*Elymus tranchycaulus* (Link) Gould ex Shinn	4×	PI 537323	**StH**	Utah, United States	PI537323L	Sun & Zhang, 2011
		PI372500	**StH**	NA	GQ855191	Wang et al., unpublished
*Elymus transhyrcanus*	4×	PI383579	**StH**	NA	GQ855193, GQ855194	Wang et al., unpublished
*Elymus violaceus* (Hormem.) Feilberg	4×	H10588	**StH**	Julianehåb, Greenland	H10588Y	Sun & Zhang, 2011
*Elymus virescens* (Piper) Gould	4×	H10584	**StH**	Julianehåb, Greenland	H10584Y, H10584K	Sun & Zhang, 2011
*Elymus virginicus*	4×	PI490361	**StH**	NA	GQ855195, GQ855196	Wang et al., unpbulished
*Elymus wawawaiensis* J. Carlson ex Barkworth	4×	PI 610984	**StH**	Washington, United States	GQ855197, EU366410	Sha et al. 2009
*Elymus wiegandii Femald*	4×	PI 531708	**StH**	Aylwin, Quebec, Canada	PI531708K	Sun & Zhang, 2011
*Hordeum arizonicum* Covas	6×	H2144	**HHH**	Mexico	GU734674, GU734675, GU734676	Wang & Sun 2011
*H. bogdanii* Wilensky	2×	H4014	**H**	Pakistan	AY137412	Petersen & Seberg, 2003
*H. brachyantherum* Nevski subsp. *brachyantherum*	4×	H2348	**HH**	U.S.A.	GU734677, GU734678	Wang &Sun 2011
*H. brachyantherum* Nevski subsp. *californicum* (Covas & Stebbins) Bothmer, N. Jacobsen& Seberg	2×	H1942	**H**	U.S.A.	AF277260	Petersen & Seberg, 2003
*H. brevisubulatum* (Trin.) Link subsp. *violaceum* Boiss. & Hohen.	2×	H315	**H**	Iran	AY137396	Petersen & Seberg, 2003
*H. bulbosum* L.	2×	H3878	**I**	Italy	AY137411	Petersen & Seberg, 2003
*H. chilense* Roem. & Schult.	2×	H1819	**H**	Chile	AY137408	Petersen & Seberg, 2003
*H. comosum* C. Presl	2×	H1181	**H**	Argentina	AY137400	Petersen & Seberg, 2003
*H. cordobense* Bothmer, N. Jacobsen & Nicora	2×	H6429	**H**	Argentina	AY137415	Petersen & Seberg, 2003
*H. depressum* (Scribn. & J. G. Sm.) Rydb.	4×	H2008	**HH**	U.S.A.	GU734670, GU734671	Wang &Sun 2011
*H. erectifilium* Bothmer, N. Jacobsen& R.B. JØrg.	2×	H1150	**H**	Argentina	AF277259	Petersen & Seberg, 2003
*H. euclaston* Steud.	2×	H1263	**H**	Argentina	AY137401	Petersen & Seberg, 2003
*H. flexuosum* Nees ex Steud.	2×	H1133	**H**	Argentina	AY137399	Petersen & Seberg, 2003
*H. fuegianum* Bothmer, Jacobsen & Jørgensen	4×	H1418	**HH**	Argentina	GU734665, GU734666	Wang & Sun 2011
*H. intercedens* Nevski	2×	H1940	**H**	U.S.A	AY137409	Petersen & Seberg, 2003
*H. jubatum* L.	4×	H2013	**HH**	Mexico	GU734672, GU734673	Wang &Sun 2011
*H. lechleri* (Steud.) Schenck	6×	H1451	**HHH**	Chile	GU734667	Wang & Sun 2011
*H. marinum* Huds. subsp. *marinum*	2×	H546	**Xa**	Spain	AY137397	Petersen & Seberg, 2003
*H. marinum* Huds. subsp. *gussoneanum* (Parl.) Thell.	2×	H299	**Xa**	Bulgaria	AF277257	Petersen & Seberg, 2003
*H. murinum* L. subsp. *glaucum* (Steud.) Tzvelev	2×	H801	**Xu**	Iran	AF277258	Petersen & Seberg, 2003
*H. muticum* J. Presl	2×	H958	**H**	Bolivia	AY137398	Petersen & Seberg, 2003
*H. parodii* Covas.	6×	H1458	**HHH**	Argentina	GU734668, GU734669	Wang & Sun 2011
*H. patagonicum* (Hauman) Covas subsp. *magellanicum* (Parodi & Nicora) Bothmer, Giles & N. Jacobsen	2×	H6209	**H**	Argentina	AY137414	Petersen & Seberg, 2003
*H. patagonicum* (Hauman) Covas subsp. *mustersii* (Nicora) Bothmer, Giles & N. Jacobsen	2×	H1358	**H**	Argentina	AY137405	Petersen and Seberg, 2003
*H. patagonicum* (Hauman) Covas subsp. *patagonicum*	2×	H1319	**H**	Argentina	AY137403	Petersen & Seberg, 2003
*H. patagonicum* (Hauman) Covas subsp. *santacrucense* (Parodi & Nicora) Bothmer, Giles & N. Jacobsen	2×	H1493	**H**	Argentina	AY137406	Petersen and Seberg, 2003
*H. patagonicum* (Hauman) Covas subsp. *setifolium* (Parodi & Nicora) Bothmer, Giles & N. Jacobsen	2×	H1357	**H**	Argentina	AY137404	Petersen & Seberg, 2003
*H. procerum* Nevski	6×	H1166	**HHH**	Argentina	GU734661, GU734662, GU734663, GU734664	Wang & Sun 2011
*H. pubiflorum* Hook. f.	2×	H1296	**H**	Argentina	AY137402	Petersen & Seberg, 2003
*H. pusillum* Nutt.	2×	H2038	**H**	New Mexico	AY137410	Petersen & Seberg, 2003
*H. roshevitzii* Bowden	2×	H7202	**H**	China	AY137416	Petersen & Seberg, 2003
*H. stenostachys* Godr.	2×	H1783	**H**	Argentina	AY137407	Petersen & Seberg, 2003
*H. tetraploidum* Covas.	4×	H6198	**HH**	Argentina	GU734679, GU734680	Wang & Sun 2011
*Lophopyrum elongatum* (Host) Á. Löve	2×	H6692	**E^e^**	Israel	AF277246	Petersen & Seberg, 2000
*Psathyrostachys fragilis* (Boiss.) Nevski subsp. *fragilis*	2×	H917	**Ns**	Iran	AF277261	Petersen & Seberg, 2000
*Psa. stoloniformis* Baden	2×	H9182	**Ns**	China	AF277264	Petersen & Seberg, 2000
*Pseudoroegneria spicata* (Pursh) Á. Löve	2×	H9082	**St**	U.S.A.	AF277245	Petersen & Seberg, 2000
*Taeniatherum caput-medusae* (L.) Nevski	2×	H10254	**Ta**	Russia	AF277249	Petersen & Seberg, 2000
*Thinopyrum bessarabicum* (Savul. & Rayss) Á. Löve	2×	H6725	**E^b^**	Russia	AF277254	Petersen & Seberg, 2000

* NA: Information not available from previous publication.

### Data Analysis

Multiple sequence alignments are made using Clustal X with default parameters [36] with manual adjustment. Phylogenetic analysis using the maximum-parsimony (MP) method is performed with the computer program PAUP* ver. 4 beta 10 [37]. All characters are specified as unweighted and unordered, and gaps are excluded in the analyses. Most-parsimonious trees are obtained by performing a heuristic search using the Tree Bisection-Reconnection (TBR) option with MulTrees on, and ten replications of random addition sequences with the stepwise addition option. Multiple parsimonious trees are combined to form a strict consensus tree. Overall character congruence is estimated by the consistency index (CI), and the retention index (RI). In order to infer the robustness of clades, bootstrap values with 1000 replications [38] are calculated by performing a heuristic search using the TBR option with MulTrees on.

In addition to maximum parsimony analysis, maximum-likelihood (ML) analysis is performed. For ML analysis, 8 nested models of sequence evolution were tested for the data set using PhyML 3.0 [39]. The general time-reversible (GTR) [40] substitution model led to a largest ML score compared to the other 7 substitution models: JC69, K80, F81, F84, HKY85, TN93 and custom (data not shown). As the result, the GTR model was used for the ML analysis. The ML analysis was performed using the Mac OS X UNIX version of GARLI v. 0.95 [41]. The runs were set for an unlimited number of generations, and automatic termination following 10,000 generations without a significant (lnL increase of 0.01) topology change. Thirty analyses were run with random starting tree topologies, and the tree with best score was used to represent gene tree. Branch support (BS) was estimated based on 100 ML bootstrap replicates in GARLI.

Nucleotide diversity was estimated by Tajima's π [42] and Watterson's θ [43] statistics. The former measure quantifies the mean percentage of nucleotide differences among all pairwise comparisons for a set of sequences, whereas the latter is simply an index of the number of segregating (polymorphic) sites. Tests of neutral evolution were performed as described by Tajima [42], and Fu and Li [44]. The above calculations were conducted by the software program DnaSP v5 [45].

## Results

Total of 87 sequences from 18 tetraploid (22 accessions) StH *Elymus*, 9 polyploid *Hordeum* species, 24 diploid *Hordeum* species/subspecies and 8 other diploid Triticeae species were analyzed. Sequence comparisons revealed five large insertions/deletions (indels). Compared to other sequences aligned here, one copy sequence (H1166s4) from hexaploid *H. procerum* and the sequence from diploid *H. cordobense* (AY134715) showed a 24 bp insertion at position 206. One sequences from *E. confusus* (PI 598463k) showed a 23 bp deletion at position 352. An 82 bp insertion was found in the sequences from polyploid species *H. fuegianum*, *H. jubatum*, and *H. tetraploidum*, and diploid *Australopyrum velutinum* and *Taeniatherum caput-medusae* as reported by Wang and Sun [35], and Petersen and Seberg [31], respectively. The sequence from *E. trachycaulus* (PI 537323L) showed a 30 bp insertion at position 1054. A 15 bp deletion was detected in the sequences from *E. canadensis* (EU366405), *E. cordilleranus* (H6486k), *E. hystrix* (EU366415), *H. arizonicum* (H2144s3), *H. brachyantherum* subsp. *californicum* (AF277260), *H. brachyantherum* subsp. *brachyantherum* (H2348s2), *H. depressum* (H2008s1) and *H. procerum* (H1166s3).

**Figure 1:**
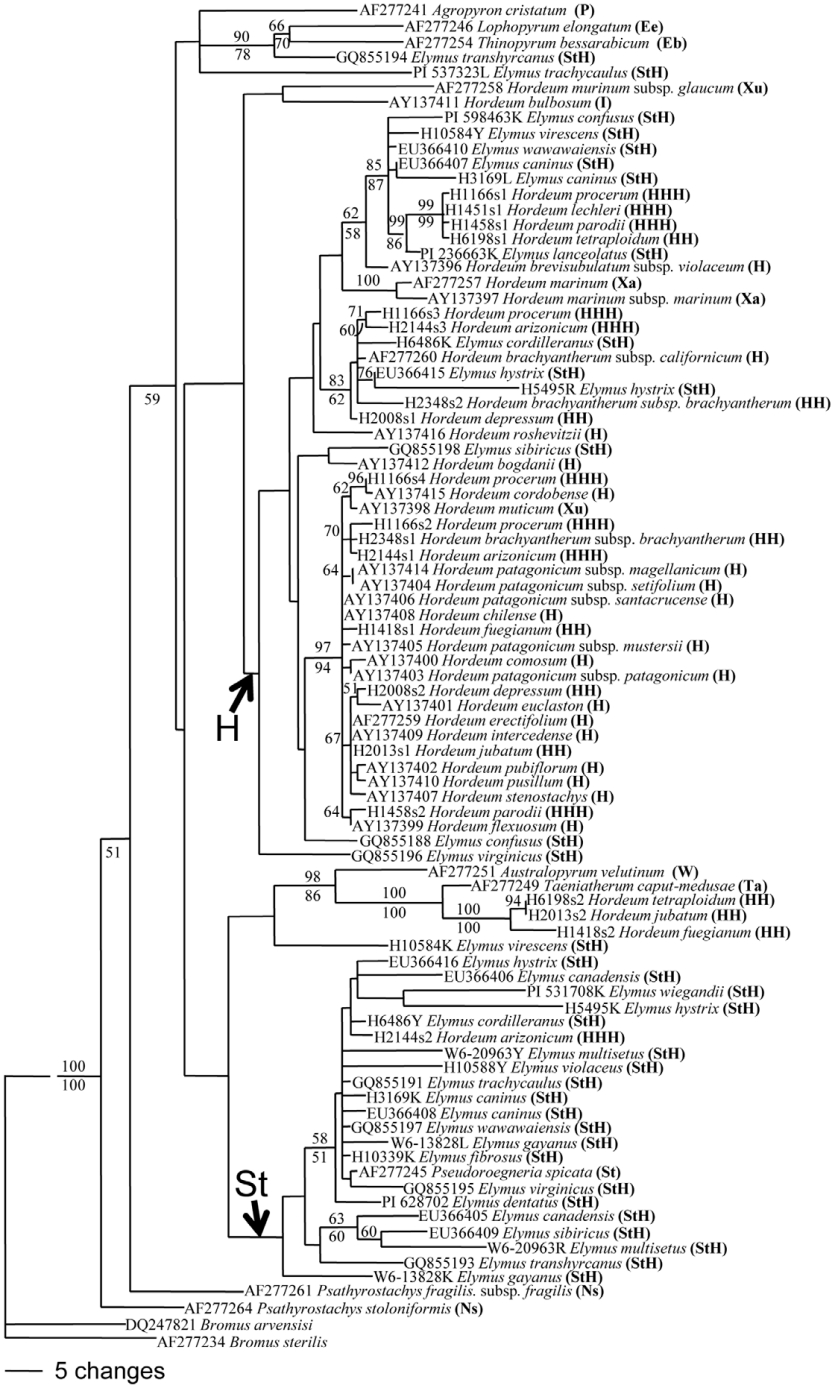
The best scoring ML tree was selected from 30 GARLI analyses under a GTR model. Numbers above branches are ML bootstrap support values, and the numbers below branches are bootstrap support values.

The 89 (including two outgroups) aligned 1221 bp *DMC* sequences showed 794 constant, 221 variable and parsimony-uninformative, and 206 parsimony-informative sites. Parsimony analysis using *Bromus arvensis* and *B. sterilis* as outgroup produced 740 equally parsimonious trees with a consistency index (CI) of 0.693, and a retention index (RI) of 0.848. Maximum likelihood analysis across 30 GARLI runs generated likelihood score ranging from –lnL6349.08703 to –lnL6355.83219. ML tree with BS is shown in Figure 1.

Two copies of sequences each from *E. caninus*, *E. cordilleranus*, *E. hystrix*, *E. sibiricus*, *E. virginicus* and *E. wawawaiensis* were well separated into two distinct groups, one grouped with the sequences from H genome, and another with St genome from *Pseudoroegneria* (Fig. 1). As unexpected, the sequence (GQ855194) from *E. transhyrcanus* formed clade with *Lophopyrum elongatum* and *Thinopyrum bessarabicum* with 90% BS in ML and 78% BS support in MP. The second copy of the sequence from *E. transhyrcanus* (GQ855193) was sister to the sequences from *E. canadensis*, *E. sibiricus* and *E. multisetus*. The sequences from polyploid *Hordeum* species fall into the clade with sequences from diploid *Hordeum* except one each from polyploid *H. arizonicum*, *H. fuegianum*, *H. jubatum*, and *H. tetraploidum* (Fig. 1). Also included in this clade are the sequences from *Elymus* species which were from the H genome. One sequence from *H. arizonicum* was clustered with the **St** genome sequences from *E. cordilleranus*, *E. hystrix*, *E. canadensis*, and *E. wiegandii*. An Asian diploid *H. brevisubulatum* subsp. *violaceum* is sister to polyploid *H. lechleri, H. parodii, H. procerum, H. tetraploidum*, *E. lanceolatus*, *E. confusus*, *E. virescens*, *E. wawawaiensis*, *E. caninus*. Within this clade, the sequence from *E. lanceolatus* is sister to the sequence from *H. lechleri, H. parodii, H. procerum, H. tetraploidum* with highly support (87% BS in ML, 85% in MP). One copy each from polyploid *H. arizonicum, H. brachyantherum* subsp. *brachyantherum, H. depressum, H. procerum, E. cordilleranus, E. hystrix* and American diploid species *H. brachyantherum* subsp. *californicum* formed a moderately supported clade (83% BS in ML, and 62% BS in MP) (Fig. 1).

Based on grouping of the sequences in phylogenetic analysis, we further separately analyzed nucleotide variation of DMC1 gene in the H genome from *Hordeum* polyploids and diploids, and *Elymus*. Some of the putative H copies of sequences from *Elymus* and *Hordeum* polyploids were not clearly put into the H clade. These sequences (PI 537323L, GQ855194 and W6-13828K from *Elymus*, H6198s2, H2013s2, H1418s2 and H2144s2 from *Hordeum*) were excluded for nucleotide diversity analysis. Estimates of nucleotide polymorphism, π and θw, were shown separately for the H genome of *Elymus*, *Hordeum* polyploid and diploid species (Table 2). The number of polymorphic sites (56) in the H genome of polyploid *Hordeum* is much lower than that (90) in its diploid donor species. The estimates of nucleotide diversity in the H genome of diploid *Hordeum* studied were θw = 0.02693, π = 0.01488. The estimates of nucleotide diversity in the H genome of polyploid *Hordeum* studied were θw = 0.0168, π = 0.01734. The number of polymorphic sites, the estimates of nucleotide diversity θw, π for the H genome in *Elymus* species was 80, 0.02774 and 0.02083, respectively. The Tajima [42], and Fu & Li's [44] tests were conducted on each data set. The Tajima, and Fu and Li's values of the diploid *Hordeum* H genome were −1.95371 (P<0.05) and −2.65118 (P<0.05), respectively, which showed significant departure from neutrality, while the Tajima, and Fu and Li's values of the polyploid *Hordeum* H genome were −0.34466 and −0.80485, respectively. The Tajima's *D* was −1.19959 and the Fu and Li's D was −1.29141 for *Elymus* H genome.

**Table 2:** Estimates of nucleotide diversity and test statistics for selection at DMC1 in polyploidy and diploid H genome.

	N	h	n	s	π	θw	Fu & Li's D	Tajima's D
*Hordeum* genome								
H (diploid)	24	21	1025	90	0.01488±0.00298	0.02693±0.00905	−2.65118*	−1.95371*
H (polyploid)	16	16	1023	56	0.01680±0.00159	0.01734±0.00652	−0.80485	−0.34466
*Elymus*								
H	12	12	1002	80	0.02083±0.00318	0.02774±0.01098	−1.29141	−1.19959

The N is the number of sequences analyzed, h is the number of haplotypes, n is the number of the sites (excluding sites with gaps/missing data), s is the number of segregating sites, π is the average pairwise diversity, θ_w_ is the diversity based on the number of segregating sites.

* : Significant at α = 0.05.

## Discussion

Origin of **H** genome in **StH**-genome *Elymus* species based on single copy nuclear gene DMC1 has previously been discussed [30]. DMC1 sequence data also showed a reticulate relationship of American polyploid species and diploid *Hordeum* [35]. However, the relationship of the H genome in polyploid *Hordeum* and *Elymus* was not previously explored.

The maximum parsimonious analysis grouped 24 sequences from *Hordeum* diploid and polyploid species together with 94% bootstrap supported value, maximum likelihood analysis also grouped these sequences together with highly supported value of 97% (Fig. 1). Only 3 *Hordeum* diploid H genome species, *H. brevisubulatum* subsp. *violaceum*, *H. brachyantherum* subsp. *californicum*, and *H. bogdanii* were grouped together with the sequences from *Elymus* H genome, indicating that the H genomes in *Elymus* originated from limited *Hordeum* diploid species, whereas the H genomes in polyploid *Hordeum* species were contributed by relative large *Hordeum* diploids. One concern is that the number of sequences from *Elymus* H genome is less than the number of sequences from *Hordeum* polyploids analyzed here, which may bias the comparison. However, phylogeny of *Elymus* StStHH allotetraploids based on three nuclear genes including relative large sample of *Elymus* species suggested that the one diploid *Hordeum* species, *H. brachyantherum* subsp. *californicum* (Syn: *H. californicum* Covas & Stebbins), is the possible H- genome donor to *Elymus* species [29], which also indicated that H genome in *Elymus* species originated from limited *Hordeum* diploid. However, the published data indicated that many *Hordeum* diploid species have contributed to the origin of polyploids in this genus, more than 10 diploid species were suggested to be the potential donors to polyploids in *Hordeum* [22], [35], [46]. Taken these together, it can be concluded that the H genomes in *Elymus* species have originated from limited gene pool, while H genomes in *Hordeum* polyploids have originated from broad gene pools.

Polyploid formation is a prominent mode of speciation in the flowering plant. Recent molecular data indicated that polyploid speciation is often more complex than initially thought [47], which is also the case in the tribe Triticeae [8], [22], [28], [29], [48], [49]. Molecular studies suggested polyploid species in many genera have multiple origins rather than single origin [1], [5], [47], [50]. It was suggested that the fates of polyploid populations of independent origins varied depending on the amount of genetic variation initially contributed by the diploid progenitors [50]. Studies have demonstrated that genetic diversity in polyploids is often similar to or higher than their diploid progenitors [47], [51], [52]. It is worth comparing the nucleotide diversities among the H genomes from *Elymus*, polyploid and diploid *Hordeum* species. This may offer an opportunity to address the potential evolutionary outcomes of polyploidization. Nucleotide diversity (π) of the DMC1 sequences from polyploid species (π = 0.02083 in *Elymus*, π = 0.01680 in polyploid *Hordeum*) is higher than that in diploid *Hordeum* (π = 0.01488). The estimates of D were significantly departure from the equilibrium neutral model at this locus in diploid *Hordeum* species (P<0.05), suggesting an excess of rare variants in diploid species. These rare variants may not contribute to the origination of polyploids. Phylogenetic analyses indeed indicated that not all diploids have contributed to the origination of polyploids in *Hordeum* and *Elymus*. Why is the genetic variation in polyploids higher than in diploid even the polyploids originated from limited number of diploid detected here? It has demonstrated that polyploidization resulted in the genome wide gene duplication which not only enables allopolyploids to tolerate more genomic variation than their progenitors, but also provides new opportunity to create functional diversification between homoeologous genes over time [52]–[54]. After gene duplicated, one of the copies may undergo mutations, if mutations are not deleterious, the mutations will not be removed by natural selection. Nucleotide diversity of this copy of gene in polyploids will be higher than that in their progenitors. Recent studies on the evolutionary rates of duplicated genes in polyploids compared to their diploid relatives showed that the evolutionary rates appear to be different among different homoeologous locus pairs [55]–[58]. Barrier *et al*. [59] found that the floral regulatory genes *APETALA1* (*ASAP1*) and *APETALA3* (*APETALA3/TM6*) are evolving much faster in the polyploid species than in the diploids. Analysis of nucleotide sequence diversity (π) of *RPB2* revealed that nucleotide diversity (π) of *RPB2* on the St genome in tetraploid *Elymus* was higher than that in the diploid *Pseudoroegneria* St genome [60].

The degree of relationships between two parents of a polyploid was suggested as a general factor affecting the amount of genomic sequence variation in allopolyploids [54]. In a study on interspecifc crosses of *Brassica* found that the overall amount of genomic change in AC (or CA) tetraploids was much lower than that in the AB (or BA) tetraploids. This was because the genetic distance between the A (*B. rapa*) and C (*B. oleracea*) was much closer than that between the A and B (*B. nigra*) [61]. A study on the timing and rate of genome variation in triticale following allopolyploidization also suggested the degree of the relationship between the parental genomes was the key factor in determining the rate of genomic sequences variation occurring during intergeneric allopolyploidization [54]. It was well demonstrated that genus *Hordeum* is monophyletic genus [21], [22], and polyploids originated from the diploid species in this genus. While the *Elymus* StH genomic species originated from the St genome donor *Pseudoroegneria* and H genome donor *Hordeum* species. The genetic distance of parental genomes in polyploid *Hordeum* is much closer than that between St and H genomes. The degree of relationships between two parents of a polyploid might be factor affecting nucleotide diversity in allopolyploids. This might explain that nucleotide diversity (π) of the DMC1 sequences in *Elymus* polyploid species (π = 0.02083) is higher than that in polyploid *Hordeum* (π = 0.01680). This speculation needs to be further studied.

One of objectives of this study is to explore the possible role of polyploid *Hordeum* species in the origin of StH *Elymus* and whether the gene flow has occurred between polyploid *Hordeum* and *Elymus* species. The *Hordeum* H genome diploids are the H genome donor to both *Elymus* StH species and polyploid species in *Hordeum*. Phylogenetic analysis revealed that the H copy of DMC1 sequence in *E. lanceolatus* is very close to the H copy of DMC1 sequence in polyploid *H. procerum*, *H. lechleri*, *H. parodii*, and *H. tetraploidum*, indicating that the H genome in *E. lanceolatus* and those four polyploid *Hordeum* species originated from the same diploid donor. Alternative explanation is that gene flow has occurred among *E. lanceolatus* and *H. procerum*, *H. lechleri*, *H. parodii*, and *H. tetraploidum*. *Elymus cordilleranus* and *E. hystrix* formed a group with ployploid *H. arizonicum*, *H. brachyantherum* subsp. *brachyantherum*, *H. depressum*, *H. procerum*, and diploid *H. brachyantherum* subsp. *californicum*. This grouping is explained either by common origin of H genome in these *Elymus* and *Hordeum* polyploids or by gene flow among these polyploids. One copy of the DMC1 sequences from North American hexaploid *H. arizonicum* was grouped with St-copy sequences from American *E. canadensis, E. hystrix, E. wiegandii* and *E. cordilleranus* (Fig. 1). The diploid *H. pusillum* and tetraploid *H. jubatum* was suggested as the parental parents for *H. arizonicum* [22], [46], [62]. cpDNA analysis suggested that *H. pusillum* could be the maternal parent of *H. arizonicum* [63]. Previous analysis of DMC1 data suggested that *H. brachyantherum* subsp. *californicum* might be one diploid genome donor of *H. arizonicum*, and the second genome donor of *H. arizonicum* might be the common ancestor of *H. brachyantherum* subsp. *brachyantherum*, and showed that one copy of DMC1 sequences of *H. arizonicum* fall outside the *Hordeum* clade of the tree [35]. DMC1 data here suggested if the St genome was not the donor species to one copy of genome in *H. arizonicum*, gene flow has occurred between *H. arizonicum* and some *Elymus* StH genome species during evolutionary process.

Analysis of β-amylase data revealed that one of the *H. jubatum* genome was placed together with *Elymus* species [29]. The role of *H. jubatum* in the *Elymus* evolutionary history has been suggested, a tetraploid similar to *H. jubatum* might have been involved in the history of *Elymus*, either through introgression between the *Elymus* and *H. jubatum*, or through a direct contribution from *H. jubatum* like species to *Elyums* [29]. Our DMC1 data showed that one of the *H. jubatum* genome with *H. tetraploidum*, *H. fuegianum*, *T. caput-medusae* and *Aust. velutinum* grouped together, *Elymus virescens* as sister to this group. Our result not only did not contradict to the suggestion that *H. jubatum* was involved at some stage in the history of StStHH *Elymus* [29], but also further expanded to that several ployploid *Hordeum* species might have involved in the evolution of StStHH *Elymus* through gene flow among them.

The study on the polyploid formation in *Tragopogon* (*Asteraceae*) indicated a lack of gene flow among polyploid plants of independent origin, even when they co-occur, suggesting potential reproductive barriers among separate lineages in polyploid species [50]. Sequence analysis of 12 nuclear loci representing 6 genes on tetraploid *Capsella bursa-pastoris* and its close diploid relative *C. rubella* showed that polyploid speciation need not result in immediate and complete reproductive isolation, and the postpolyploidization hybridization and introgression can contribute significantly to genetic variation in newly formed polyploid [64]. Molecular characterization of a diagnostic DNA marker for domesticated tetraploid wheat indicated gene flow from wild tetraploid wheat to hexaploid wheat [65]. Our results suggested that gene flow among different polyploids in Triticeae species might play an important role in polyploid speciation and evolution.
